# A highly-sensitive anti-Müllerian hormone assay improves analysis of ovarian function following chemotherapy for early breast cancer^☆^

**DOI:** 10.1016/j.ejca.2014.06.011

**Published:** 2014-09

**Authors:** Joyce Chai, A. Forbes Howie, David A. Cameron, Richard A. Anderson

**Affiliations:** a MRC Centre for Reproductive Health, Queens Medical Research Institute, University of Edinburgh, Edinburgh, UK; b Edinburgh Breast Unit and Edinburgh University Cancer Research Centre, Western General Hospital, Edinburgh, UK

**Keywords:** AMH, Breast cancer, Ovarian function, Chemotherapy, Ovarian reserve

## Abstract

**Aim:**

Anti-Müllerian hormone (AMH) shows promise as a biomarker of the ovarian reserve but current assays are insufficiently sensitive to allow assessment of this post-chemotherapy in most women. We have assessed a new highly sensitive AMH assay (Ansh picoAMH) in the evaluation of ovarian activity in women with very low ovarian reserve after chemotherapy.

**Methods:**

A prospective cohort and an independent validation cohort of premenopausal women with early breast cancer (eBC) were recruited at the time of diagnosis (combined *n* = 98), and ovarian reserve markers 2–5 years later following chemotherapy were assessed in relation to menstrual activity.

**Results:**

The picoAMH assay had a limit of detection of 7.5 pg/ml. AMH clearly distinguished women with ongoing menses from those with amenorrhoea at 2 years after diagnosis (mean 522 ± 169 versus 8.9 ± 1.3 pg/ml, *P* < 0.0001) with high predictive value for continuing menses or amenorrhoea for the subsequent 3 years. AMH was detectable in more women than using a previous assay (*P* = 0.004). Other markers of the ovarian reserve (follicle-stimulating hormone (FSH), inhibin B) were also of discriminatory value but to lesser extents. This finding was validated in a second, independent cohort of women treated for eBC.

**Conclusion:**

The 10-fold increased assay sensitivity showed very clear distinction between groups based on ovarian activity with excellent prediction of future menses or amenorrhoea. This will improve assessment of post-chemotherapy ovarian function in women and may aid treatment decisions.

## 1 Introduction

Adjuvant chemotherapy is a mainstay of treatment of early breast cancer (eBC) as it results in an improvement in both disease-free and overall survival rate [1]. Breast cancer survivors in their reproductive age are now confronted with the long-term consequences of exposure to these treatments, and therefore quality of life issues including fertility are becoming more important.

Chemotherapeutic agents can have a profound impact on ovarian function. They cause a depletion of the follicle pool in a drug- and dose-dependent manner [2]. Many women become amenorrhoeic during or after chemotherapy and chemotherapy-related amenorrhoea (CRA) is often seen as a sign of ovarian failure [3], [4]. Recovery of menses in some women after chemotherapy illustrates the limitation of amenorrhoea as a marker of complete loss of the ovarian reserve; conversely the resumption of menses may be only brief indicating preservation of only a very low ovarian reserve.

Anti-Müllerian hormone (AMH) is becoming established as a biochemical marker of ovarian reserve [5], [6], [7]. It has been shown to be more sensitive than inhibin B and follicle-stimulating hormone (FSH) and ultrasound markers antral follicle count (AFC) and ovarian volume in assessing chemotherapy-induced ovarian follicle loss [8]. AMH concentrations showed a rapid and marked fall during chemotherapy, with undetectable concentrations in many women after chemotherapy using the currently available assays [8], [9], [10], [11], [12], [13]. AMH is also undetectable for several years before the menopause in normal women [14], [15], [16], [17]. Thus current assays are unable to detect AMH in women with very low ovarian reserve, inadequately distinguishing such women from those without the likelihood of future ovarian activity. The diagnosis of ovarian failure has important therapeutic implications as well as for fertility, sexual health and non-reproductive e.g. bone health [18], [19], [20], thus its early and reliable diagnosis is central to the ongoing management of women with breast and other cancers. Endocrine therapy is often used after chemotherapy in women with eBC and may complicate interpretation of conventional diagnosis of ovarian failure, thus markers of ovarian function which are more independent of such therapies are required [18].

The main objective of this study was to investigate the value of a highly sensitive AMH assay in the assessment of ovarian function after chemotherapy in women with eBC, in comparison with other current markers of the ovarian reserve.

## 2 Patients and methods

Two cohorts of premenopausal women with eBC were recruited from the Edinburgh Breast Unit to a 5-year prospective study of ovarian function (*n* = 56 and *n* = 42, respectively). All had operable breast cancer and reported regular menses in the absence of hormonal contraception. Patients were recruited to the study after giving informed consent, usually before undergoing definitive loco-regional surgery and adjuvant chemotherapy for their breast cancer. The first cohort was used to assess the main study questions, and the second for validation. Chemotherapy regimens for these women have been previously described and consisted of sequential anthracycline-CMF or anthracyclines and taxanes in most [21], [22] with 14 women in cohort 1 not given any chemotherapy; therapeutic decisions were not influenced by this study. Both studies received Ethics Committee approval. Patient characteristics are shown in Table 1. The proportions of women receiving endocrine therapy did not differ between groups in either cohort.

**Table 1:** Characteristics of women in original cohort (cohort 1) and the validation cohort (cohort 2).

	Number in group	Age (years)	Endocrine therapy (*n*)
*Cohort 1*			
Overall	53	42.5 ± 0.9	45
Ongoing menses	10	35 ± 1.4⁎	6
Transient amenorrhoea	4	41 ± 2.9	3
Amenorrhoea	25	44.2 ± 0.9	23
No chemotherapy	14	45.8 ± 1.4	13

*Cohort 2*			
Overall	42	43.0 ± 0.9	34
Ongoing menses	8	40.2 ± 1.6	6
Transient amenorrhoea	3	32.3 ± 3.1	2
Amenorrhoea	31	44.9 ± 0.7	26

Data are mean ± sem.

Endocrine therapy consisted of tamoxifen, with additional goserelin in 15 (with one woman receiving only goserelin). Three women received anastrozole.

⁎ *P* < 0.05 versus both amenorrhoea and no chemotherapy groups.

Patients were evaluated at 2, 3, 4, and 5 years after initial diagnosis of breast cancer in cohort 1, and after 1 and 3 years in cohort 2. Visits were scheduled to be in the early follicular phase (D2-5) if appropriate. Menstrual data were coded as amenorrhoea when there was no menstrual bleeding in the preceding 6 months, as transient amenorrhoea when there was return of menses after a period of amenorrhoea, or as having ongoing menses throughout treatment up till the time of analysis.

AMH was assayed using the Ansh labs pico-AMH ELISA kit (Ansh Catalog no. AL-124, Webster, TX). For comparison with previous assays, serum samples taken at 2 years in cohort 1 also analysed using the Active MIS/AMH ELSIA (Beckman Coulter, Chaska, MN), sensitivity 0.05 ng/ml [21]. FSH was measured by enzyme-linked immunosorbent assay (ELISA) [23], and both intra-assay and inter-assay coefficients of variation were less than 8%. Inhibin B was measured as previously described [24], with sensitivity 7.8 pg/ml; intra-assay and inter-assay coefficients of variation were less than 5% and 8%, respectively. Estradiol was measured by ELISA (Demeditec, Kiel-Wellsee, Germany) with coefficients of variation <8%.

### 2.1 Statistical analyses

Data are presented as mean ± sem. The primary analysis was comparison of hormone concentrations versus menstrual function at 2 years post diagnosis, with groups compared by ANOVA; in longitudinal analyses timepoints were compared to data at 2 years using Dunn’s posthoc test. Proportions of women were compared using Fisher’s exact test. All analyses were performed using SPSS (version 20). A two-tailed value of *P* < 0.05 was considered statistically significant.

## 3 Results

### 3.1 Assay characteristics

The lower limit of detection (LLOD: the lowest concentration of AMH in a sample that can be detected with a 95% probability) was determined by serial dilution of pooled patient serum in two different assay plates. The LLOD was 7.5 pg/ml, in agreement with the manufacturer’s quoted range of 1.7–11.6 pg/ml. Inter-assay analytical variation (CV) was 5.6% at 67.7 pg/ml and 2.0% at 185 pg/ml. Intra-assay analytical variation was less than 10% from 25 pg/ml to 783 pg/ml.

### 3.2 Menses after chemotherapy

Amongst the 56 women recruited to the initial cohort, 39 in the chemotherapy group and 14 in the non-chemotherapy group remained in the study at 2 years, the key time point for analysis; the remaining three women had either withdrawn from the study because of disease recurrence or had discontinued follow-up after hysterectomy and oophorectomy. The majority of patients enrolled in this study received a combination chemotherapy regimen that incorporated cyclophosphamide, an anthracycline and a taxane, as previously described [21]. The 39 women who had chemotherapy were subdivided into three groups according to their menstrual pattern over the 2–5 year followup period: ongoing menses (*n* = 10), transient amenorrhoea (*n* = 4), and amenorrhoea (*n* = 25). Ages of these groups are shown in Table 1. Women with ongoing menses were younger than women who were amenorrhoeic (*P* < 0.05) and the no chemotherapy group (*P* < 0.05); the age of the transient amenorrhoea group was not significantly different to the others.

### 3.3 Reproductive hormones after chemotherapy

**Fig. 1:**
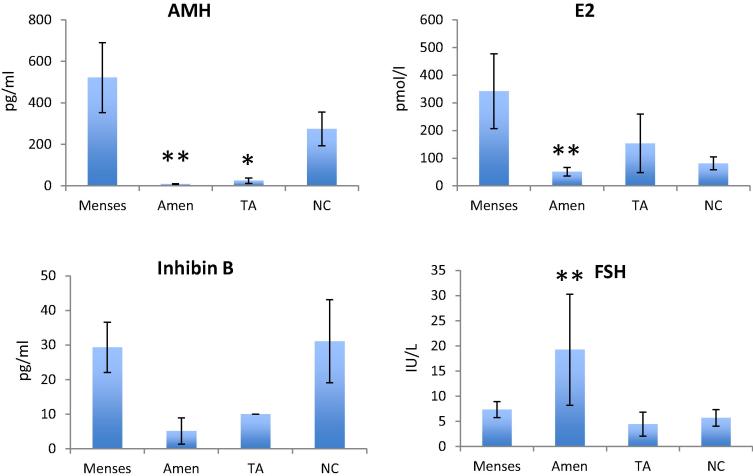
Serum concentrations of anti-Müllerian hormone (AMH), estradiol, inhibin B and follicle-stimulating hormone (FSH) in four groups of women at 2 years after diagnosis of breast cancer. The patient groups were women with on-going menses (*n* = 10), amenorrhoea (Amen, *n* = 25), transient amenorrhoea (TA, *n* = 4) and those who had not received chemotherapy (NC, *n* = 14). ^∗^*P* < 0.05; ^∗∗^*P* < 0.001 versus group with ongoing menses. Mean ± sem.

Serum concentrations of all hormones (AMH, inhibin B, estradiol, and FSH) at 2 years by menstrual function are shown in Fig. 1.

#### 3.3.1 Anti-Müllerian hormone

**Fig. 2:**
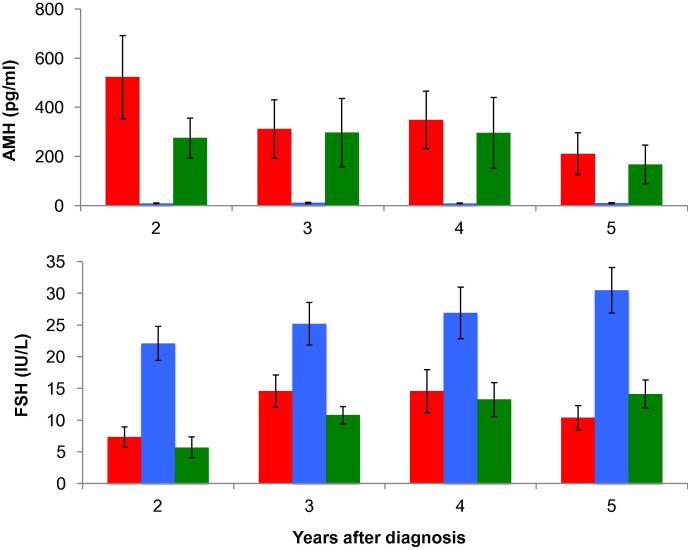
Anti-Müllerian hormone (AMH) and follicle-stimulating hormone (FSH) in women with ongoing menses (red), with amenorrhoea (blue) and in the no chemotherapy group (green) at 2, 3, 4 and 5 years after diagnosis. There were no significant changes in AMH in any of the three groups over time. There was a significant rise in FSH in the no chemotherapy group over time (*P* = 0.02), but no significant change in the ongoing menses or amenorrhoea groups. Mean ± sem.

AMH clearly discriminated between groups being detectable in all women with ongoing menses, and undetectable in most women with amenorrhoea (Fig. 1), mean at 2 years 522 ± 169 [range 12–1425 pg/ml] versus 8.9 ± 1.3 pg/ml [all <39.6 pg/ml], *P* < 0.0001. This marked difference was sustained with very similar data at 3, 4 and 5 years with a non-significant decline in AMH through follow-up (Fig. 2). Overall, AMH was undetectable in only one woman at a single time point in those with continuing menses, whereas it was undetectable in 81 of 100 samples in the amenorrhoeic group. Mean AMH was also higher in the ongoing menses group than in the transient amenorrhoea group (*P* < 0.05) but was not different to the no chemotherapy group (*P* < 0.05). In the groups with ongoing menses and the no chemotherapy group (i.e. excluding the two amenorrhoeic groups), the rate of detection of AMH in women using the picoAMH assay was higher than using a previous generation assay (20 versus 12 of 24 women; *P* = 0.004).

Women whose menses returned after a period of amenorrhoea showed a low but variable pattern of AMH concentrations. Two women with undetectable AMH at 2 years showed some recovery during followup, to concentrations of 55 and 63 pg/ml at 5 years. One of the women with detectable AMH at 2 years showed a fluctuating pattern, with AMH becoming undetectable at 3 and 4 years, but a higher value at 5 years (145 pg/ml versus 61 pg/ml at 2 years). In the other woman AMH was close to the limit of detection at 3 years and was undetectable at 4 and 5 years.

#### 3.3.2 Inhibin B

Inhibin B concentrations were very low throughout the period of investigation in all groups (Fig. 1) and while it tended to be higher in women with continuing menses, it was undetectable in 5 of 10 of them and there were no significant differences between groups.

#### 3.3.3 Estradiol

Estradiol concentrations mirrored menstrual pattern, separating women with and without menses (*P* < 0.0001; Fig. 1).

The transient amenorrhoea group, although small, showed comparable patterns to that seen with AMH. One woman with initially detectable then declining AMH showed a similar profile for E2, and a woman with initially undetectable but then rising AMH had parallel changes in E2. Women in the no chemotherapy group showed fluctuating E2 concentrations, which were not significantly different from either the ongoing menses or amenorrhoeic groups, nor did they vary significantly with time.

#### 3.3.4 Follicle-stimulating hormone

FSH concentrations were markedly higher in women with persistent amenorrhoea than in the group with ongoing menses (*P* = 0.0001; Fig. 1). These differences were observed at all time points up to 5 years, with no significant changes in FSH concentrations in either of those two groups over that period (Fig. 2). The transient amenorrhoea group showed low FSH concentrations at 2 years but marked variability (without statistically significant differences) at subsequent time points. The no chemotherapy group showed similar FSH concentrations to the ongoing menses group at 2 years but showed a significant rise with time (*P* = 0.02).

### 3.4 Discriminatory value of ovarian markers

**Fig. 3:**
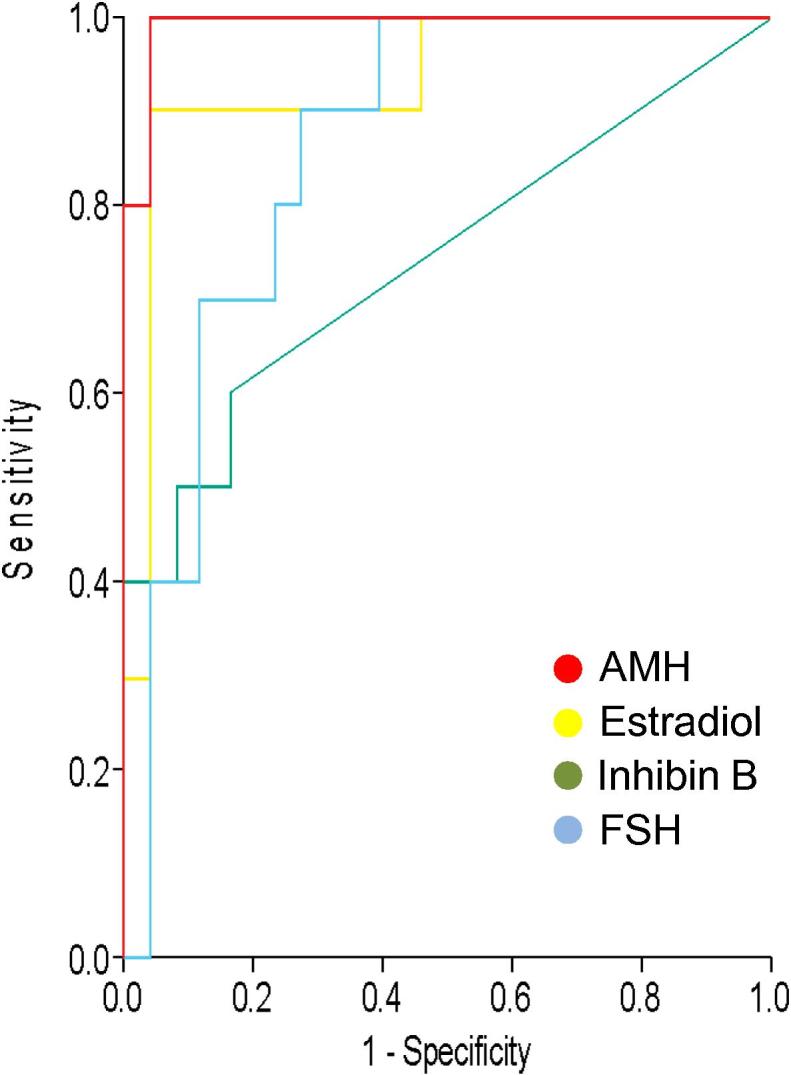
Receiver operator characteristic (ROC) curve analysis of anti-Müllerian hormone (AMH), estradiol, inhibin B and follicle-stimulating hormone (FSH) in the discrimination of women with and without amenorrhoea. Areas under the curve (AUC) were AMH: 0.99, estradiol: 0.93; inhibin: B 0.74 and FSH: 0.86.

The ability of the various hormones to discriminate between women with and without menses was assessed by receiver operator characteristic (ROC) curve analysis using data at 2 years (Fig. 3). These gave areas under the curve (AUC) values of 0.99 for AMH (95% confidence intervals (CI) 0.97–1.01, *P* < 0.0001, peak likelihood ratio (LR) 9.6), 0.93 for E2 (CI 0.83–1.03, *P* < 0.0001, peak LR 9.6), 0.74 for inhibin B (CI 0.54–0.94, *P* = 0.03, peak LR 2.1) and 0.86 for FSH (CI 0.73–0.98, *P* = 0.001, peak LR 7.2). The optimum cut-off for AMH was 16.1 pg/ml, giving a sensitivity of 96% and specificity of 90%. As this value is close to the limit of detection of the assay, this result means that a woman with detectable AMH using this assay is very likely to have ongoing menses for at least 3 years thereafter whereas a woman with undetectable AMH does indeed have no remaining ovarian reserve and will remain amenorrhoeic.

Including data from the women with transient amenorrhoea with those with ongoing menses only slightly reduced the AUC for AMH to 0.97 (peak LR 6.7) and for E2 to 0.86 (both *P* < 0.001), whereas the AUC for inhibin B was reduced to 0.65 and became not significant; AUC for FSH increased slightly to 0.87 (*P* = 0.0001).

### 3.5 Validation cohort analysis

These findings were assessed in a second cohort of 42 women with eBC following treatment with chemotherapy followed up for 3 years as described above. 31 were amenorrhoeic by 1 year and remained so, eight showed continuing menses at 1 year, and 3 were amenorrhoeic at 1 year but had recovered menses by 3 years (Table 1).

**Fig. 4:**
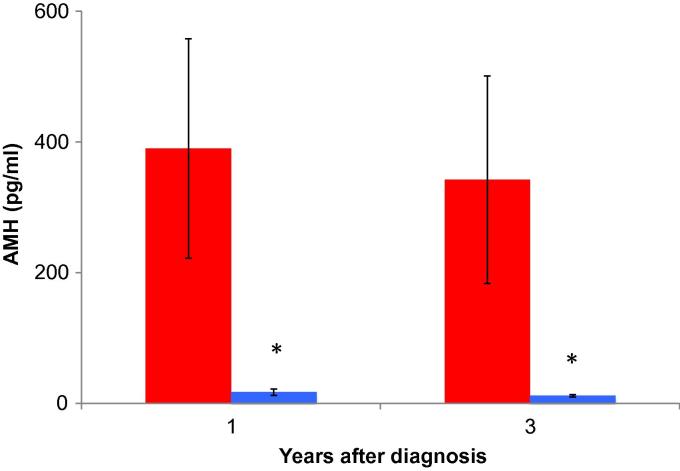
Anti-Müllerian hormone (AMH) values at 1 and 3 years after diagnosis in the validation cohort of women treated with chemotherapy for early breast cancer. Red, those with ongoing menses (*n* = 8); blue, those who were amenorrhoeic (*n* = 34). ^∗^*P* < 0.001 versus group with ongoing menses. Mean ± sem.

AMH concentrations were markedly different in the two groups, mean values 20-fold higher in the women with ongoing menses than in the amenorrhoea group (*P* = 0.0001; Fig. 4). AMH was detectable in all women with menses at 1 year and remained so at 3 years; while it was close to the limit of detection in two women (<20 pg/ml) both had higher concentrations at 3 years (46 and 155 pg/ml) indicating ongoing recovery from the effect of chemotherapy. In contrast, AMH was undetectable in 23 of the 31 amenorrhoeic women at 1 year and very low (<20 pg/ml) in a further 4.

Three women showed return of menses between 1 and 3 years of followup. All had detectable AMH concentrations at 1 year, and showed marked rises in AMH at 3 years (29–567, 750–1405 and 58–357 pg/ml) although these levels remained very low considering these women’s age (38, 28 and 29 years, respectively). Overall mean AMH in women with menses at 3 years was higher than in the same women at 1 year (558 ± 196 versus 354 ± 170 pg/ml, *P* = 0.02, *n* = 8).

## 4 Discussion

In this prospective study, we examined a panel of reproductive hormones of ovarian function including AMH, inhibin B, FSH, and estradiol in eBC survivors, and used a highly sensitive AMH assay to evaluate the ovarian reserve in this group of patients. We demonstrate significant differences in AMH, FSH and estradiol between women with amenorrhoea compared with those who continued to menstruate at 2–5 years after diagnosis. This is concordant in results with previous data in which AMH, FSH and estradiol were found to differ between menstruating and amenorrhoeic women at 1–4 years after chemotherapy [25] although the proportions of women with undetectable AMH were not shown. That study also suggested a significant difference in inhibin B level, not confirmed in the present data. These data thus confirm the value AMH as a marker of ovarian reserve in this context.

This study also evaluated the predictive potential of these markers for determining future menstrual status. AMH was consistently detectable in those with ongoing menses and had the highest predictive value by ROC analysis. An undetectable level of AMH using this ultrasensitive assay at 2 years followup suggests that menstruation is unlikely to resume whereas a detectable AMH level suggests that ongoing menstruation is very likely for at least 3 years thereafter. This contrasts with this and previous studies using less sensitive assays which have identified populations of women with evidence of ovarian function but undetectable AMH [26], [27]. For those women who are pre-menopausal at diagnosis of an oestrogen receptor (ER) positive breast cancer, this information would be very helpful in the clinic. There is good evidence that the sequence of an aromatase inhibitor after tamoxifen gives superior outcomes to continuing with tamoxifen, but in women with functioning ovaries, aromatase inhibitors are ineffective so there is always concern about switching such patients [28], [29]. Therefore patients could be treated with tamoxifen initially after completion of chemotherapy, and if at 2 years still had detectable AMH, a switch to an aromatase inhibitor would not be appropriate, whereas it would be for those with undetectable AMH concentrations. The lack of effect of tamoxifen on AMH [30] supports this approach. Women who show recovery of menses after amenorrhoea are an important group in this context, although they were infrequent in both cohorts studied here and including them with those with ongoing menses made little difference to the discriminatory ability of AMH. They had variably detectable AMH concentrations, and larger groups of these women are needed to assess the value of AMH in them.

A substantial proportion of young women retain ovarian activity after modern adjuvant chemotherapy for early stage breast cancer [4]. Menstruation is a poor indicator of fertility potential, but the limited data indicate that women who retain ovarian function after chemotherapy can often conceive [31], [32], probably reflecting their young age. In normal young women, low AMH is not associated with reduced fecundity [33]. Their reproductive lifespan may however be markedly reduced, and AMH may be of value to detect that and predict remaining duration of potential fertility. In women with ongoing menstruation AMH was generally very low (522 ± 169 pg/ml at 2 years) for their age (mean 35 years), and similar to the no-chemotherapy group whose mean age was 45 years. These data highlight the risk of early menopause even in those with ongoing menses several years after chemotherapy [34]; such women may also be at increased risk of post treatment infertility [35]. There are a paucity of data linking post-treatment AMH to the clinically important variables of reproductive lifespan and fertility but the development of new assays able to detect low levels of AMH may allow further research to explore these relationships.

The present analyses are limited particularly by the numbers of patients included, and by the inclusion only of women with breast cancer. The number of women who showed recovery of menses after amenorrhoea was small and it would be valuable to increase this group although this is a feature of the age related incidence of breast cancer and the current use of alkylating agent based chemotherapy. It is unclear whether this approach will be of value in relation to other diagnoses and treatments.

In conclusion, these data demonstrate the value of a more sensitive AMH assay in assessing ovarian function after chemotherapy, and predicting ovarian activity over the following years. The improved sensitivity of this assay also highlights the profound loss of ovarian reserve in these women. Although these data require further validation, the measurement of AMH post chemotherapy may offer valuable information to both patients and their clinicians.

## Conflict of interest statement

RAA has undertaken consultancy work for Beckman Coulter and Roche Diagnostics.
